# Editorial: Innovative translational research to identify colorectal cancer biomarkers for personalized medicine

**DOI:** 10.3389/fgene.2024.1414214

**Published:** 2024-05-23

**Authors:** Sergi Castellví-Bel, Beatriz Carvalho

**Affiliations:** ^1^ Gastroenterology Deparment, Fundació de Recerca Clínic Barcelona-Institut d’Investigacions Biomèdiques August Pi i Sunyer, Centro de Investigación Biomédica en Red de Enfermedades Hepáticas y Digestivas, Clínic Barcelona, University of Barcelona, Barcelona, Spain; ^2^ Department of Pathology, Netherlands Cancer Institute, Amsterdam, Netherlands

**Keywords:** colorectal cancer, biomarker, prevention, personalised medicine, translational research

Colorectal cancer (CRC) is the third most common cancer worldwide and ranks second, after lung cancer, as a cause of cancer-related death. Over 1.9 million people develop CRC each year, and the incidence of this tumor is increasing (Morgan et al., 2023).

CRC is a high-prevalence disease which continues to have relatively high mortality and no simple avoidable cause. CRC is preventable by detecting and removing adenomas via sigmoidoscopy or colonoscopy screening. Due to the invasive nature and cost of endoscopy, intermediate screening methods, such as fecal occult blood test (FOBT), are used to select patients for colonoscopy. However, there is still the need to identify CRC biomarkers that will improve screening, early detection, and disease follow-up, and attain better tumor profiling, state-of-the-art functional characterization of genetic variants, and new therapy approaches for this disease (Online resource 1).

This Research Topic in Frontiers in Genetics includes six research articles describing work using innovative translational research to identify colorectal cancer biomarkers for personalized medicine that will improve screening, early detection, and the disease follow-up, and attain better tumor profiling, state-of-the-art functional characterization of genetic variants, and new therapy approaches.

In the article by Pinto et al. and Marinkovic et al., the authors contributed to the characterization of the phenotype of *NTHL1* tumor syndrome, an autosomal recessive rare disease caused by biallelic inactivating variants in the *NTHL1* gene. They studied 467 patients, including 228 patients with colorectal polyposis and 239 patients with a familial/personal history of multiple tumors. Altogether, they identified nine affected patients with polyposis with biallelic pathogenic or likely pathogenic *NTHL1* variants, as well as two index patients with one pathogenic or likely pathogenic *NTHL1* variant in concomitance with a missense variant of uncertain significance. Their findings indicated that *NTHL1* tumor syndrome is a multi-tumor syndrome strongly associated with polyposis and not with multiple tumors without polyposis.

The article by Stanojevic et al. analyzed the role of *MTHFR* C677T and A1298C single-nucleotide polymorphisms in the early detection and response to neoadjuvant chemoradiotherapy in locally advanced rectal cancer. The *MTHFR* 677C and 1298A alleles were identified as low-penetrance risk factors for rectal cancer, while no correlation was obtained in terms of response to treatment. Data produced in this study point to low-cost, non-invasive, and easily determined factors that might help identify specific subgroups of patients that should be monitored more closely. This is especially important in developing countries and can be used for the construction of genetic cancer risk prediction panels.

The fecal DNA test has emerged as a non-invasive alternative for CRC screening in the average-risk population. There is insufficient evidence in China to demonstrate the effectiveness of this approach. Liu et al. and Danisik et al. performed a large-scale study for CRC screening in Wuhan, Hubei province, China. A total of 98,683 subjects aged between 45 and 60 years were screened by a fecal DNA test for determining the methylation status. Participants who tested positive were advised to undergo colonoscopy. In total, 4,449 (4.5%) subjects tested positive, and 3,200 (71.9%) underwent colonoscopy. Among these, 2,347 (73.3%) had abnormal colonoscopy findings, of which 1,330 (56.7%) subjects received a pathological diagnosis. Detection rates for CRC and advanced precancerous lesions were 1.3% and 2.3%, respectively. Detection rates for nonadvanced adenomas and polyps were 14% and 21.6%, respectively. In conclusion, preliminary real-world evidence suggested that fecal DNA tests had a promising diagnostic yield in population-based CRC screening in China.

The article by Marinkovic et al. and Liu et al. explored predictive biomarkers for neoadjuvant chemoradiotherapy response in locally advanced rectal cancer. Through transcriptomics analysis and qRT-PCR, genes associated with treatment response were identified. Among 75 patients, responders (46.6%) were defined by a clinical complete response or favorable histopathological tumor regression grading (TRG 1-2). Non-responders (53.4%) had higher extramural vascular invasion on MRI. Notably, hematological parameters like the neutrophil-to-monocyte ratio and specific blood cell counts showed significant differences between responder and non-responder groups. Logistic regression highlighted tumor morphology and hematological parameters as predictive factors, suggesting a potential for routine clinical use.

Cancer treatment effectiveness is hampered by drug resistance. Capecitabine and irinotecan are commonly used therapeutic agents in the treatment of CRC patients. In the article by Danisik et al. and Stanojevic et al., the underlying clonal dynamics of resistance to these agents using the high-resolution barcode technology and identification of effective second-line drugs were studied in Caco-2 and HT-29 cell lines. Their results revealed clonal dynamics of irinotecan and capecitabine formed by both pre-existing and *de novo* barcodes. Collateral drug sensitivity revealed drugs that were effective alone and in combination. The use of barcoding technology unwrapped the clonal dynamics of chemotherapy-induced drug resistance not only from harvested cell populations but also from longitudinal sampling throughout the course of clonal evolution.

The article by Li et al. and Pinto et al. focused on cancer immunology and immunotherapy, which are fundamental areas in CRC research and treatment currently. The authors aimed to establish immune-related gene prognostic indices (IRGPIs) for prognosis evaluation in CRC, retrieving expression and clinical data from TCGA and GEO databases, and immune-related gene data from the ImmPort and InnateDB databases. They identified 49 immune-related genes associated with the prognosis of CRC, 17 of which were selected for an IRGPI. The IRGPI model significantly differentiated survival rates of CRC patients in the different groups. This study established a promising immune-related risk model for predicting survival in CRC patients. This could help in better understanding the correlation between immunity and the prognosis of CRC, providing a new perspective for personalized treatment of CRC.

## Author’s note

Online resource 1: Biomarkers for colorectal cancer. https://www.youtube.com/watch?v=cGR0QOqCBp4. Learn the basics about biomarkers and CRC, why testing is important, and what learning about your biomarker mutations can mean for your treatment options and cancer outcomes. Visit the Colorectal Cancer Alliance website to learn more at https://colorectalcancer.org/treatment/types-treatment/why-biomarkers-matter.
